# Association between exposure to pesticides and brain cancer in adults: a systematic review

**DOI:** 10.47626/1679-4435-2026-1575

**Published:** 2026-09-22

**Authors:** Mariana Rangel Vieira, Beatriz Ramos Lucente, Murilo Luciano Langoni, Herling Gregorio Aguilar Alonzo

**Affiliations:** 1 Universidade Estadual de Campinas, Faculdade de Ciências Médicas, Campinas, SP, Brazil

**Keywords:** brain neoplasms, pesticides, adult, carcinogenesis.

## Abstract

This systematic review aimed to critically evaluate the association between pesticide exposure and the brain tumors in adults, taking into account epidemiological evidence, biologically plausible mechanisms, and public health implications. A systematic search of international databases, including PubMed, SciELO, Embase, and MEDLINE, was conducted for the period from January 2012 through January 2025, using predefined inclusion and exclusion criteria, with the inclusion of epidemiological studies and systematic reviews. The available evidence indicated associations between exposure to specific pesticide classes, particularly carbamates and organophosphates, and increased brain tumor risk, especially for gliomas and meningiomas, with relevant findings reported in large studies such as AGRIculture & CANcer (AGRICAN) and CERENAT. Proposed biological mechanisms supporting the plausibility of these associations include oxidative stress, genetic damage, epigenetic alterations, and chronic inflammation. However, important methodological limitations remain, particularly regarding exposure assessment and adequate control for confounding. Taken together, these findings suggest that prolonged occupational exposure to certain pesticides may be associated with an increased risk of central nervous system tumors, highlighting the need for preventive measures, more stringent regulatory oversight, and further studies incorporating more accurate exposure assessment methods.

## INTRODUCTION

Currently, the global incidence of brain cancers ranges from 3.5 to 4.6 cases per 100,000 population, corresponding to approximately 321,000 to 330,000 new cases annually [^1^]. Incidence increases progressively with age, although some types, such as medulloblastomas, are more common in children. Geographically, high-income countries have higher detection rates, whereas low-income countries face challenges related to underdiagnosis [^2^].

Mortality associated with this form of cancer is quite high, totaling 227,000 to 248,000 deaths per year, with mortality rates approaching incidence rates (2.6 to 3.1 per 100,000 population) [^1^]. Among patients who survive, brain cancer is frequently associated with severe neurological sequelae, with a significant impact on quality of life of patients and their families. Neurological deficits vary according to the affected region. For example, tumors in the dominant hemisphere frequently present with speech disorders, whereas tumors in the nondominant hemisphere may cause spatial distortions and constructional apraxia [^3^].

Among the factors associated with the development of these tumors, exposure to ionizing radiation, particularly during childhood, constitutes the most well-established environmental risk factor for brain tumors [^4^]. Moreover, frequent occupational exposure to organic solvents, heavy metals, and polycyclic aromatic hydrocarbons has also been identified as a potential risk factor [^5^]. Additional reported risk factors include specific genetic variants, such as the EGF 61-A polymorphism in Caucasian populations, processed meat consumption, personal use of hair dyes, and pesticide exposure [^6^].

With regard to pesticide exposure, a study published in 2021 found a 13% increase in the risk of brain cancer morbidity and mortality among farmers compared with nonfarmers [^7^]. This finding is even more relevant in Brazil, which ranks among the world’s largest consumers of pesticides and has experienced increasing pesticide use over recent decades, particularly in the South and Southeast regions [^8^]. A study conducted from 1996 to 2010 compared brain cancer mortality rates in two regions of the state of Rio de Janeiro and showed a 3.8% increase in the agricultural Serrana region, while no significant percent changes were observed in the metropolitan region [^9^].

The effects of pesticides are also thought to be cumulative; that is, their carcinogenic potential may increase with longer duration of exposure. In recent years, the medical literature has identified pesticide exposure as one of the main environmental factors associated with the development of central nervous system (CNS) tumors [^10^]. However, the magnitude of this risk varies depending on the type of exposure, the toxicity profile of the pesticide involved, and the histological subtype of the tumor [^7^]. Further studies are thus needed to better understand the causal relationships and underlying pathophysiological mechanisms, as well to quantify the associated risks and integrate the evidence, which remains fragmented across the literature.

Therefore, this study aimed to analyze recent evidence by synthesizing and discussing its findings to improve understanding of the association between pesticide exposure and the occurrence of different types of brain cancer in adults.

## METHODS

This study aimed to investigate whether occupational or environmental exposure to pesticides is associated with an increased risk of brain cancer in adults compared with no exposure. A systematic literature search was conducted for articles published from January 2012 to January 2025, using combinations of the following keywords: humans, cancer, brain cancer, chronic effect, pesticides, types of use - insecticides, herbicides, and fungicides. Searches were performed in PubMed, SciELO, BVS, Embase, LILACS, and MEDLINE. Articles published in English, French, Spanish, and Portuguese were selected in accordance with the PRISMA (Preferred Reporting Items for Systematic reviews and Meta-Analyses) guidelines.

Inclusion criteria: cross-sectional, case-control, cohort, and ecologic studies, as well as meta-analyses, published within the specified period and available in the previously described databases were eligible for inclusion. Studies were required to investigate the association between pesticide exposure and different subtypes of CNS tumors.

Exclusion criteria: studies employing designs other than those specified in the inclusion criteria were excluded, as were studies published before January 2012 or after January 2025. Studies deemed to have low reliability due to limitations in methodological design, study conduct, or interpretation of the results that reduced the strength of the resulting evidence obtained were also excluded.

**Figure 1:**
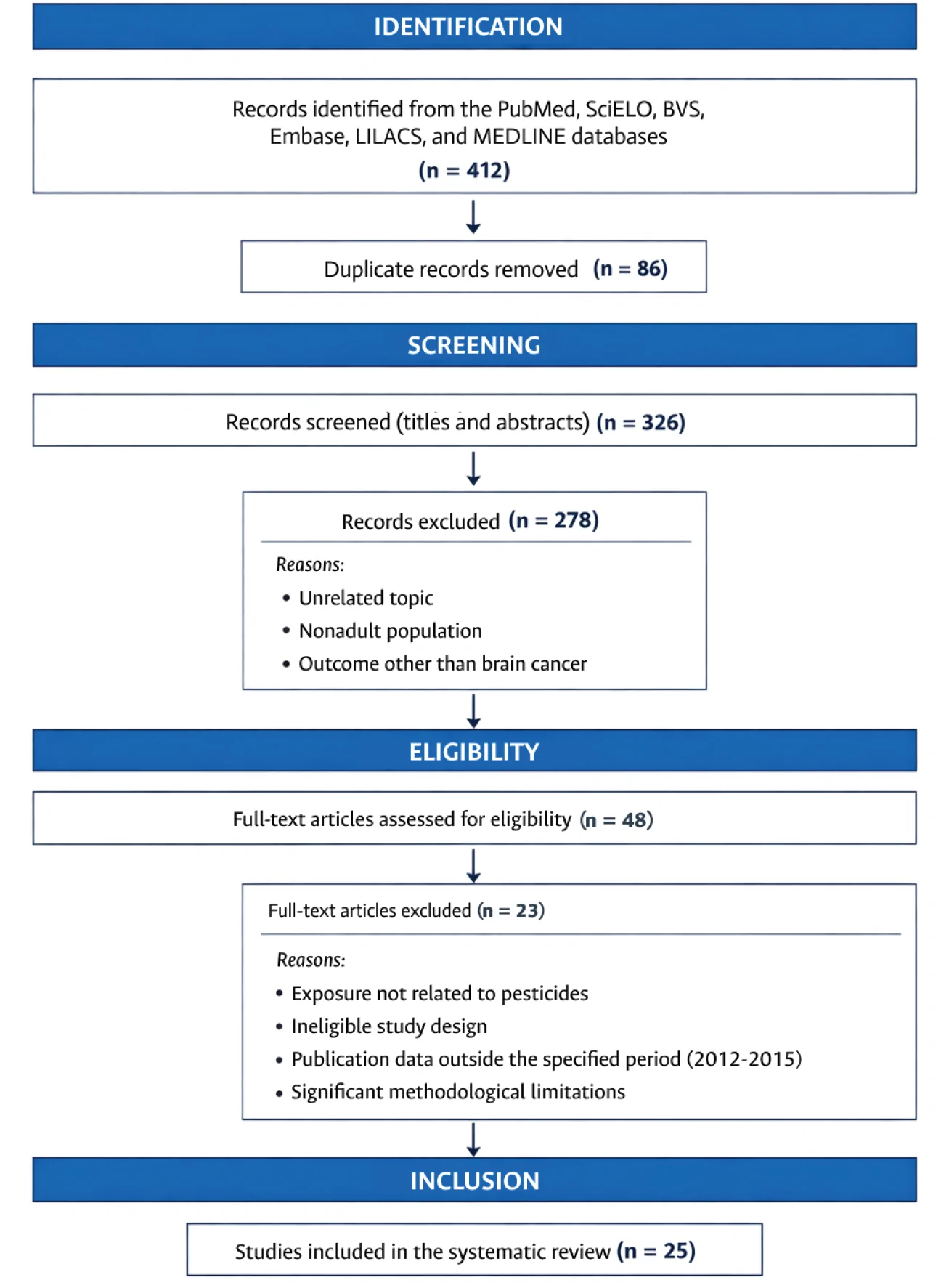
Flow diagram of the study selection process.

Study selection and data assessment: the initial screening was conducted by reviewing article abstracts and excluding studies that did not meet the inclusion criteria, particularly those that did not directly address the topics of interest. Potentially relevant articles subsequently underwent full-text assessment. Extracted data included publication year, study country/region, study design, evaluated population, type of pesticide investigated, exposure type (occupational, environmental, or mixed), associated brain tumor type, and main findings. To enhance methodological rigor, study quality was assessed according to criteria encompassing relevance, methodological clarity, adequacy of sample size, and consistence of the reported findings (Figure 1).

## RESULTS AND DISCUSSION

Recent medical literature examining the association between pesticide exposure and brain cancer in adults includes systematic reviews, meta-analyses, prospective cohort studies (eg, AGRICAN), and multicenter case-control studies (eg, CERENAT and Upper Midwest Health Study), with large European agricultural cohorts and mechanistic reviews being particularly prominent.

A total of 25 articles were included in this systematic review, ensuring broad geographic coverage, with studies from France, the United States, Italy, and Brazil, as well as multisite studies and reviews of global scope. The included studies encompass different pesticide classes (organochlorines, organophosphates, carbamates, fungicides, and herbicides), exposed populations (farmers, pesticide applicators, and individuals residing near agricultural areas), and histological subtypes of CNS tumors, including gliomas and meningiomas. Most studies employed regression models adjusted for relevant confounders, such as age, sex, smoking, and educational attainment, as well as specific exposure matrices to estimate cumulative exposure. Exposure assessment methods included occupational classification (farmer or pesticide applicator), self-reported pesticide use, residential proximity to agricultural areas, and, in some studies, biomarker measurements, such as pesticide residues or metabolites in tissue samples.

Evidence of pesticide exposure and brain cancer suggests a modest yet consistent association in some contexts between pesticide exposure and an increased risk of CNS tumors. Recent studies with advanced analytical techniques, such as gas chromatography-mass spectrometry combined with chemometric analysis, have detected pesticide metabolites in human brain tissue samples and identified exposure-related patterns associated with tumor risk in relatively small sample series. Although such studies do not establish causality, they provide direct evidence of intracranial pesticide exposure or bioaccumulation in patients with brain tumors, thereby supporting the biological plausibility of associations reported in epidemiological studies and experimental models [^11^].

Overall, meta-analyses reported a modest overall increase of approximately 13% in the risk of CNS tumors associated with agricultural activities [^7^], while transnational prospective cohort studies provided more robust and specific evidence of associations between particular pesticide classes and certain brain tumor subtypes [^12^]. In the AGRICAN cohort, carbamate use was associated with a higher incidence of CNS tumors, with risk tending to increase with longer duration of exposure to specific compounds. These findings highlight carbamates, in particular, as potential candidates for further investigation [^12^]. Increased risks for some tumor subtypes, such as gliomas and meningiomas, were also observed among farmers who used pesticides, with variations according to crop and livestock type, pesticide application method [^13^], and residential proximity to pesticide-treated agricultural areas [^14^].

With regard to these tumor subtypes, several studies have suggested differences between gliomas and meningiomas, both of which have been frequently evaluated. Some compounds exhibit different magnitudes of association with these tumor types, suggesting that distinct etiological pathways may be involved [^15^]. In contrast, a case-control study conducted in the Midwestern United States found no association between pesticide exposure and glioma development; however, this study has several limitations [^16^].

Furthermore, multicenter case-control studies, including CERENAT, yielded heterogeneous findings: some reported positive associations between occupational pesticide exposure and specific CNS tumors, whereas no significant associations were observed in other subgroups and strata. These findings suggest that risk may vary according to the chemical class involved, exposure intensity and duration, and individual susceptibility [^17^].

Toxicological reviews and experimental studies have identified multiple mechanisms that support the biological plausibility of an association between pesticide exposure and brain tumor development (Charts 1 and 2).

**Chart 1:** Tumor subtype and association with pesticide classes

Tumor subtype	Associated pesticide class	Main findings	References
Glioma	Carbamates and organophosphates	Positive association with carbamate use (AGRICAN) and exposure to certain organophosphates (case-control studies). Positive association with consumption of fruits and vegetables with levels of pesticides residues (two U.S. cohorts). Risk varies according to exposure duration.	^7^, ^13^, ^18^
Meningioma	Persistent exposure to carbamates and organophosphates	Indications of an association with carbamates in agricultural cohorts. Earlier studies suggest a possible association with organochlorines, potentially related to bioaccumulation.	^10^, ^12^, ^19^
Other rare subtypes	Limited or inconsistent evidence	Tumors such as medulloblastomas and astrocytomas are less common in adults, with no consistent evidence linking these tumors to pesticide exposure in adults.	^7^, ^17^

AGRICAN = AGRIculture & CANcer.

**Chart 2:** Biological mechanisms involved in pesticide-related carcinogenesis

Biological mechanism	Description	References
Oxidative stress and DNA damage	Generation of reactive oxygen species, oxidative DNA damage, and lipid peroxidation, leading to potentially carcinogenic mutations. Mechanism is associated with exposure to organochlorines and organophosphates.	^20^
Genetic damage and alterations in the cell cycle	Alterations in cell cycle checkpoints, DNA double-strand breaks, and modulation of tumor suppressor pathways, including p53 and p21. In vivo and in vitro evidence indicates that these alterations may be associated with pesticide exposure.	^21^
Epigenetic alterations	Tumor suppressor gene hypermethylation and modulation of histones and microRNAs induced by pesticide exposure, particularly organochlorines with recent epigenetic alterations observed following past exposure.	^20^
Chronic inflammation and immune dysfunction	Chronic pesticide exposure may be associated with the establishment of an inflammatory microenvironment in the central nervous system, promoting neoplastic cell proliferation and survival.	^19^
Endocrine disruption and neuromodulation	Neurotoxic effects of certain pesticides and endocrine disruption, with alterations in pathways involved in the proliferation, apoptosis, and survival of neuronal and glial cells.	^19^

Recent mechanistic revies underscore carcinogenesis is unlikely to involve a single pathway and instead probably results from multiple genotoxic, epigenetic, and metabolic events together with gene-environment interactions [^7^]. Comprehensive toxicological reviews further emphasize that pesticides differ substantially in their toxicokinetic properties, including lipophilicity and the resulting accumulation of organochlorines in tissues such as adipose tissue and the placenta), as well as in their toxicodynamics, toxicity, environmental fate, and carcinogenic potential. Accordingly, broad generalizations may be inaccurate unless the specific substance is taken into account [^19^].

Several methodological limitations and potential sources of bias may contribute to the heterogeneity across studies. A major source of inconsistency is exposure assessment, as many studies have relied on occupation, self-reported pesticide use, or residential proximity as proxies for exposure, while quantitative historical biomarker data are available in only a few studies [^22^]. Latency and duration of follow-up are additional concerns because brain tumors have a long latency period, and short follow-up periods may underestimate associations [^7^]. Exposure to complex mixtures also poses a challenge, as workers are rarely exposed to a single chemical agent. Furthermore, residual or inadequately controlled confounding by factors such as radiation exposure, viral infections, educational attainment, socioeconomic status, and other occupational exposures may hinder the attribution of observed associations to specific compounds. Finally, the relative rarity of brain tumors results in few income events and, together with limited sample sizes, reduced statistical power to estimate risks associated with less commonly used pesticides [^7^,^15^,^19^].

Thus, the European Food Safety Authority and comprehensive evidence assessment highlight the need for higher-quality epidemiological evidence, including standardized methods for exposure assessment, harmonized exposure classification, the use of biomarkers, and adequate control for confounding, to enable more robust causal inference [^22^].

Considering the available body of evidence and its implications for health promotion, the adoption of public health practices and policies targeting the following areas is recommended:

a) Policy and regulation: adoption of precautionary principle for pesticides with suggestive and consistent evidence of carcinogenicity or procarcinogenic mechanisms, particularly persistent compounds; regulatory reviews based on the overall body of evidence to guide reassessment of authorized uses and occupational exposure limits [^7^]; and strengthening of toxicological assessments by incorporating subcellular endpoints, such as epigenetic alterations and biomarkers of DNA damage, as well as studies of pesticide mixtures.
b) Occupational protection: reduction of worker exposure through decreased pesticide use and, when warranted by the available evidence, restrictions or bans of specific products; preferential use of less toxic products; appropriate use of personal protective equipment, with adequate oversight during application; use of closed application systems whenever feasible; and implementation of protocols for decontamination and removal of contaminated clothing. These measures are especially important for professional pesticide applicators, for whom evidence has shown more consistent associations [^12^].
c) Surveillance and monitoring: routine implementation of comprehensive health surveillance for exposed populations, including biomonitoring of agricultural workers through the measurement of key metabolites in blood and urine and the incorporation of biomarkers of oxidative stress and DNA damage into surveillance studies. The detection of metabolites in tumor tissues supports the potential value of biomonitoring [^23^] and reinforces the need to further investigate associations between pesticide exposure and different malignancies [^24^].
d) Public health and education: implementation of national and local guidelines aimed at protecting communities living in or near agricultural areas and reducing residential pesticide exposure. Recommended measures include preventing contaminated work clothing from being brought into the home, establishing buffer zones between pesticide application areas and residences, and providing information on periods of increased risk of spray drift. Residential proximity studies suggest that populations living near pesticide-treated areas may be at increased risk [^25^].
e) Research and prioritization: future studies should prioritize improved exposure characterization using sales and application records, sensors, standardized questionnaires, and historical biomarkers. Well-powered longitudinal studies are needed to assess rare tumor subtypes, with detailed exposure assessment according to pesticide class (eg, carbamates, organochlorines, organophosphates, and fungicides), given the benefits observed in AGRICAN when analyses were stratified by type of use and activity [^10^]. Translational mechanistic studies linking biomarker-based measures of human exposure to epigenetic and signaling pathway alterations in brain tissue are also needed to strengthen causal inference [^20^].

## CONCLUSIONS

The current body of evidence indicates a modest increased risk of brain tumors associated with agricultural activities and occupational exposure to pesticides, despite considerable heterogeneity across studies. Associations appear to be more consistent for certain pesticide classes, particularly carbamates, and for specific tumor subtypes. Toxicological evidence, together with the detection of pesticide metabolites in tumor tissues, further supports the biological plausibility that certain pesticides may contribute to brain carcinogenesis. However, methodological heterogeneity and limitations in exposure assessment preclude broadly generalizable conclusions. Further research using objective exposure measures, pesticide-specific analysis, prospective study designs, and biomarkers is therefore needed to strengthen causal inference and more accurately characterize risks. Nonetheless, the available evidence supports preventive measures to enhance worker protection and biomonitoring, reduce occupational exposure, and inform regulatory reassessments.

## Data Availability

The data supporting the findings of this study are available within the article.
